# Prediction of Human Papillomavirus (HPV) Association of Oropharyngeal Cancer (OPC) Using Radiomics: The Impact of the Variation of CT Scanner

**DOI:** 10.3390/cancers13092269

**Published:** 2021-05-08

**Authors:** Reza Reiazi, Colin Arrowsmith, Mattea Welch, Farnoosh Abbas-Aghababazadeh, Christopher Eeles, Tony Tadic, Andrew J. Hope, Scott V. Bratman, Benjamin Haibe-Kains

**Affiliations:** 1 Radiation Medicine Program, Princess Margaret Cancer Centre, University Health Network, Toronto, ON M5G 2CI, Canada; Reza.reiazi@uhnresearch.ca (R.R.); carrowsm@universityhealthnetwork.onmicrosoft.com (C.A.); mattea.welch@rmp.uhn.ca (M.W.); farnoosh.abbasaghababazadeh@uhnresearch.ca (F.A.-A.); Christopher.eeles@uhn.ca (C.E.); tony.tadic@rmp.uhn.ca (T.T.); andrew.hope@rmp.uhn.ca (A.J.H.); scott.bratman@rmp.uhn.ca (S.V.B.); 2 Department of Medical Biophysics, University of Toronto, Toronto, ON M5J 1L7, Canada; 3 Department of Radiation Oncology, University of Toronto, Toronto, ON M5T 1P5, Canada; 4 Ontario Institute for Cancer Research, Toronto, ON M5G 0A3, Canada; 5 Department of Computer Science, University of Toronto, Toronto, ON M5T 3A1, Canada; 6 Vector Institute, Toronto, ON M5G 1M1, Canada

**Keywords:** radiomics, computed tomography, robustness, human papillomavirus, oropharyngeal cancer

## Abstract

**Simple Summary:**

Recent studies exploring the application of radiomics features in medicine have shown promising results. However, variation in imaging parameters may impact the robustness of these features. Feature robustness may then in turn affect the prediction performance of the machine learning models built upon these features. While numerous studies have tested feature robustness against a variety of imaging parameters, the extent to which feature robustness affects predictions remains unclear. A particularly notable application of radiomics in clinical oncology is the prediction of Human Papillomavirus (HPV) association in Oropharyngeal cancer. In this study we explore how CT scanner type affects the performance of radiomics features for HPV association prediction and highlight the need to implement precautionary approaches so as to minimize this effect.

**Abstract:**

Studies have shown that radiomic features are sensitive to the variability of imaging parameters (e.g., scanner models), and one of the major challenges in these studies lies in improving the robustness of quantitative features against the variations in imaging datasets from multi-center studies. Here, we assess the impact of scanner choice on computed tomography (CT)-derived radiomic features to predict the association of oropharyngeal squamous cell carcinoma with human papillomavirus (HPV). This experiment was performed on CT image datasets acquired from two different scanner manufacturers. We demonstrate strong scanner dependency by developing a machine learning model to classify HPV status from radiological images. These experiments reveal the effect of scanner manufacturer on the robustness of radiomic features, and the extent of this dependency is reflected in the performance of HPV prediction models. The results of this study highlight the importance of implementing an appropriate approach to reducing the impact of imaging parameters on radiomic features and consequently on the machine learning models, without removing features which are deemed non-robust but may contain learning information.

## 1. Introduction

Recent advances in radiomics, the process of extracting descriptors from radiological images by mathematical algorithms, have led to a large set of quantitative imaging features becoming available to both research and clinical communities. A number of radiomics-driven computer models have shown promising results for personalized medicine, especially in oncological applications [1,2,3,4]. Radiomic features exhibit different levels of complexity, and express properties of lesion shape and voxel intensity histograms, as well as the spatial arrangement of intensity values at the voxel level (texture). They can be extracted either directly from the images or after applying different filters or transformations [5,6,7].

However, the introduction of radiomics into clinical practice has been lacking. This is largely due to low reproducibility, caused by variation in imaging parameters [8] and segmentation (intra observer variability) [9], which affects classifier performance and is of paramount importance in ensuring the successful application of radiomics to the field of oncology [10,11]. The effects of variability in image acquisition on the robustness of radiomic features have been found to be greater than that of segmentation [12] and inter-observer variability [13]. Consequently, conclusions regarding the performance of radiomic models must be treated with caution [14] since the results are vulnerable to image acquisition variability [15].

A prediction task that has received broad attention in the literature is the prediction of human papillomavirus (HPV)-associated oropharyngeal cancer (OPC) from radiological images [16,17,18,19,20]. HPV-positive OPC is now recognized as a distinct disease, with implications for treatment and prognosis [21,22]. HPV status is currently ascertained from tumor tissue using immunohistochemistry to visualize expression of the p16 protein, or by using in situ hybridization for viral DNA. As such, standard HPV testing is invasive as it requires tissue sampling. Therefore, seeking a non-invasive yet accurate way to assess HPV status is an important research goal. Recently, a statistical radiomics approach analyzing Computed Tomography (CT) images has emerged as a potential non-invasive approach to predicting HPV status in OPC patients [16,17,19,23]. Despite recent improvements [23], the predictive performance of these models is still limited. One possible reason for this deficiency is vulnerability to variation in imaging parameters. Therefore, as radiomics is used to predict HPV status, it is important to assess the impact of the imaging parameters, e.g., scanner type, on predictions.

In this study, we evaluated the impact of imaging domain attributable to the CT scanner typeon the prediction of human papillomavirus (HPV) association of oropharyngeal cancer (OPC) using radiomics models. We leveraged a large image database compiled consecutively from treated OPC patients at the Princess Margaret Cancer Centre with the aim of assessing the influence of scanner manufacturer on feature reproducibility and the prediction of HPV status. We found that the scanner manufacturer affects the prediction of HPV status by machine learning models built onCT-derived radiomic features. Our results also indicate that robust features might reduce overfitting in radiomic models and subsequently affect the accuracy of the prediction.

## 2. Methods

**Figure 1:**
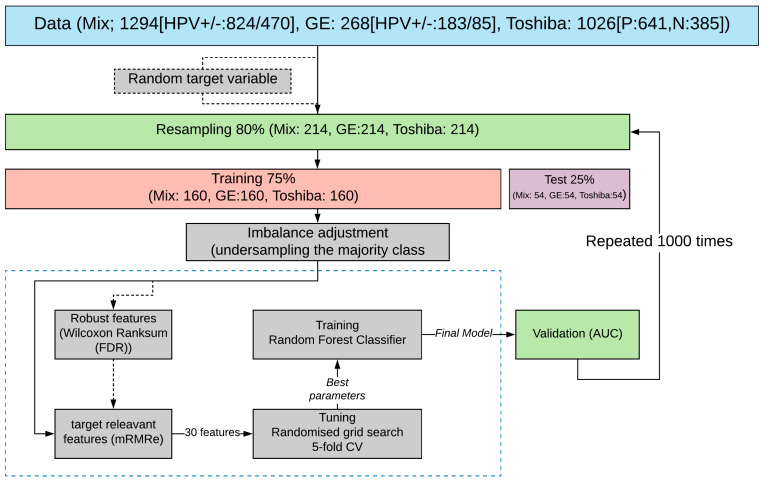
Schematic diagram of the research methodology. Downstream processes are as follows: sampling original patient cohort, train and test set splitting, class imbalance adjustment followed by selecting robust (Wilcoxon rank-sum) and HPV-relevant features (mRMRe), and finally model validation by estimating AUC values over the test set. The overall process is repeated 1000 times (also w/random variables) to evaluate the statistical significance of the reported values.

The schematic overview of this study is shown in Figure 1.

### 2.1. Dataset

Patient data were retrospectively (2006–2016) collected from the Princess Margaret Cancer Centre University Health Network and were approved by the institutional review board (REB 17-5871). All experiments were performed in accordance with the relevant guidelines and regulations of the institution. The primary patient cohort in this paper was collected by consecutively searching the institutional database for in-patients who met the following criteria: (1) had Oropharyngeal cancer (OPC); and (2) had completed p16 immunohistochemistry. In total, we analyzed CT images from 1294 OPC patients with known HPV status determined by p16 immunohistochemistry (Figure S1). Mean patient age was 61 years ± 10.5 (standard deviation). HPV status was positive in 824 patients (641 Toshiba and 183 GE) and negative in 470 patients (385 Toshiba and 85 GE). Distribution of HPV status was almost the same in the two groups (+HPV: 0.78[Toshiba]/0.22[GE]; −HPV: 0.81[Toshiba]/0.19[GE]). Intravenous contrast was used in 371 patients (all from the Toshiba scanner). The dataset was subsequently stratified by CT scanner manufacturer (Toshiba, GE, and both (Mix)). Next, the following nine configurations of train–test sets were made: (1) Toshiba–Toshiba, (2) GE–GE, (3) Toshiba–GE, (4) GE–Toshiba, (5) Mix–Mix, (6) Toshiba–Mix, (7) GE–Mix, (8) Mix–Toshiba, and (9) Mix–GE. The Mix group contained the same number of samples from two scanner manufacturers (Toshiba and GE). The first and second terms of each configuration represent the scanner type (i.e., Toshiba, GE or Mix) of the train and test sets respectively.

### 2.2. Feature Extraction

For each patient, the primary gross tumor volume (GTV) was contoured by the treating oncologist (single observer). Prior to extraction, images were resampled to 1 × 1 × 1 mm voxels and the intensities were normalized with a bin width of 25 Hounsfield units (HU). We extracted a total of 1874 radiomic features from each patient’s manually segmented GTV using PyRadiomics (version 3) [24]. The extracted features belong to six feature classes. This includes Shape features describing the shape and geometric properties of the region of interest (ROI) such as volume, maximum diameter along different orthogonal directions, maximum surface, tumor compactness, and sphericity. First-order statistics features describe the distribution of individual voxel values without concern for spatial relationships. These are histogram-based properties reporting the mean, median, maximum, and minimum values of the voxel intensities on the image, as well as their skewness (asymmetry), kurtosis (flatness), uniformity, and randomness (entropy). Second-order statistics features include the so-called textural features [25], which are obtained by calculating the statistical inter-relationships between neighboring voxels. They provide a measure of the spatial arrangement of voxel intensities, and hence of intra-lesion heterogeneity. Such features can be derived from the grey level co-occurrence matrix, quantifying the incidence of voxels with the same intensities at a predetermined distance along a fixed direction, or from the grey level run–length matrix quantifying consecutive voxels with the same intensity along fixed directions [26]. Feature breakdown according to the group they belong to is as follows: 14 Shape, 320 GLRLM (Gray Level Run Length Matrix) and GLSZM (Gray Level Size Zone Matrix), 360 FO (First Order Statistics), 480 GLCM (Gray Level Co-Occurrence Matrix), 280 GLDM (Gray Level Dependence Matrix) and 100 NGTDM (Neighboring Gray Tone Difference Matrix).

Features are also obtained after mathematically transforming the images through the application of imaging filters, with the aim of identifying repetitive or non-repetitive patterns, suppressing noise, and highlighting details. These filters include wavelet transforms, square, square root, gradient, exponential, and Laplacian transforms of Gaussian [27]. Further explanation about the details of the aforementioned filters can be found in PyRadiomics documentation. The distribution of features based on the imaging filter is as follows: Original (unfiltered images) 88, Exponential, Gradient, Square and Square-root each 88; Local Binary Pattern (lbp) and Laplacian of Gaussian (LoG) each 264; and Wavelet 704. Finally, all the radiomic features were scaled by subtracting the median and dividing by the interquartile (the range between the 1st quartile and the 3rd quartile).

### 2.3. Data Sampling and Splitting

Figure 1 shows the overall workflow of this study. Initially, 80% of the data was resampled without replacement and then was split into train and test sets in the proportion of 75/25. The remaining 20% was held out for final validation. Subsequently, the training set was used for feature selection (discussed later) and model training, and the resultant model was tested on the test set. The above process was repeated 1000 times to evaluate the statistical significance of the obtained results. The median value of the obtained performance metric is reported in Figure 1.

### 2.4. Reproducibility Analysis and Feature Selection

T-Distributed Stochastic Neighbor Embedding (t-SNE) clustering was applied to visualize potential scanner dependencies in the radiomic features. t-SNE is a non-linear technique for dimensionality reduction that is particularly well suited to the visualization of high-dimensional datasets. The algorithm starts by calculating the probability of similarity between points in high-dimensional space, and then tries to present these similarities as distances for a meaningful representation of data points in lower-dimensional space. We test whether the distribution of observations obtained between the two different groups on selected variables are systematically different using the Wilcoxon rank–sum test. Our assumption was that features with the same distributions across two scanner manufacturers will have the least scanner dependency (we define these features as “robust” if their association with scanner manufacturer is not statistically significant). We corrected the *p*-values for tests and computed the false discovery rate (FDR) using Bonferroni correction [28] with a threshold set at 5% for significant dependency.

### 2.5. Feature Selection

In order to select relevant features for HPV prediction, we used the Minimum Redundancy, Maximum Relevance (mRMR) Ensemble Feature Selection (mRMRe) implemented in the PymRMRe package (version 1.0.4) [29]. This technique is a feature selection approach that selects the features with a high correlation with the class (maximum relevance) and a low correlation between themselves (minimum redundancy). We used the F-statistic to calculate the correlation with the class (relevance) and the Pearson correlation coefficient to calculate the correlation between features (redundancy).

### 2.6. Tuning and Training

Imbalance adjustment was done by under-sampling the majority class (HPV positive), and a Random forest (RF) classifier was trained to predict HPV status (Figure 1). We used the GridSearchCV function in Scikit-learn (0.23.2) for exhaustive searches over the specified values of the model’s hyper-parameter such as the number of trees, maximum depth of the tree, and the minimum number of samples required to be at a leaf node. Each model was trained on the 1000 features selected by mRMRe. Finally, RF models were trained with and without robust features. The predictive performance of the HPV status classifiers were assessed by calculating the area under the curve (AUC) (i.e., the area under the curve of receiver operating characteristics). For training, five-fold cross-validation was applied in which training sets were randomly partitioned into five groups. One group was used for testing, and the other groups were retained for training. For each combination, the training–testing procedures were repeated 100 times until each sample in the data set was assigned a prediction score. The final AUC was estimated based on the average prediction score (1000 times). In parallel, all the above processes were repeated by replacing actual target labels with random binary labels to compare the result with random models.

## 3. Results

**Figure 2:**
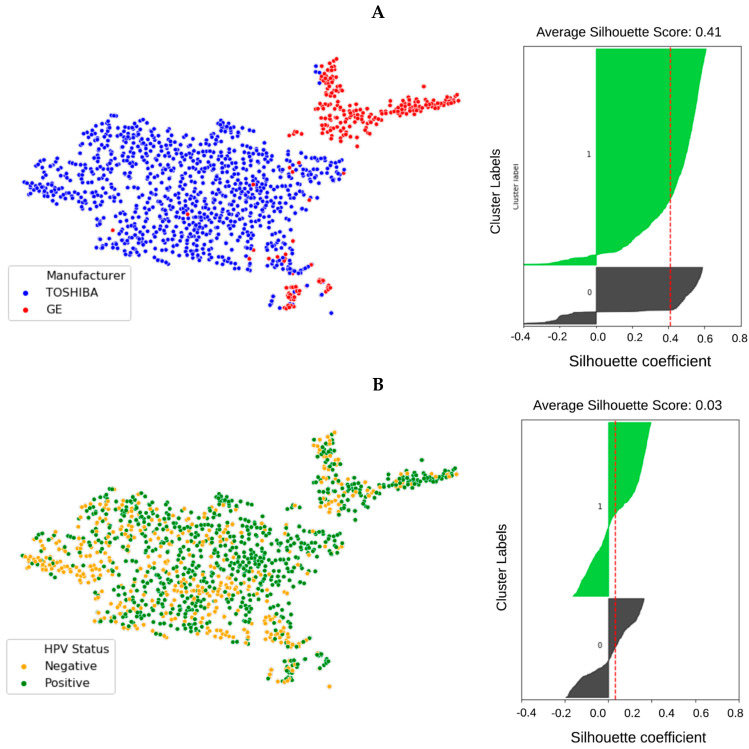
t-SNE clusters labeled by scanner manufacturer ((**A**) red: GE, blue: Toshiba) and the samples’ HPV status ((**B**) orange: HPV negative, green: HPV positive). The corresponding silhouette analysis and average silhouette score is shown on the right. The impact of scanner manufacturer is clearly seen when samples are labeled by manufacturer type. However, radiomic features do not show intrinsic dependency on the sample’s HPV status.

**Figure 3:**
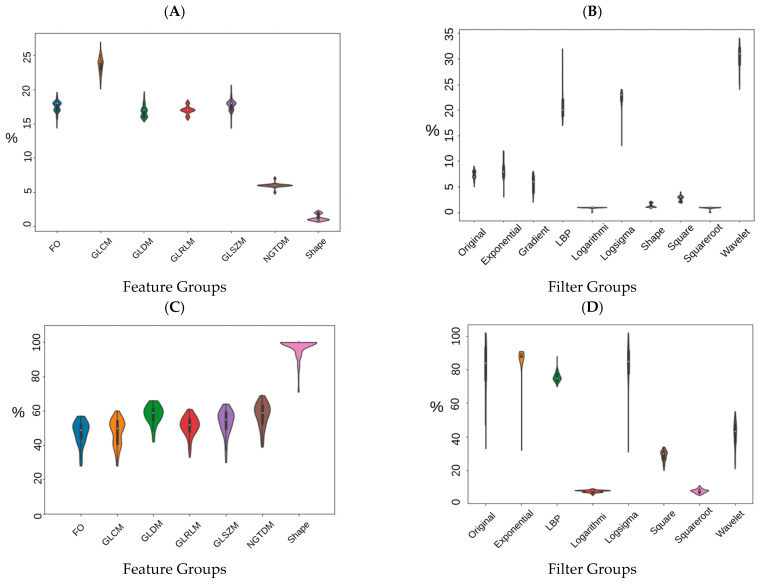
Percentage of robust features according to the type of feature group (**A**,**C**) and imaging filters (**B**,**D**). (**A**,**B**) have been normalized to the total number of robust features and (**C**,**D**) have been normalized to the number of features in each feature group (**C**,**D**).

In order to visualize the distribution of scanner manufacturers in high-dimensional feature space, we performed t-SNE dimensionality reduction directly on the scaled features, plus a silhouette analysis for all samples. Cases have been labelled with the type of scanner manufacturer (Figure 2A). Clustering showed considerably higher dependency on the scanner manufacturer (average Silhouette score ~0.4) than HPV status (average Silhouette score ~0.03) when all radiomics features were used. We also labelled the clustered data with the HPV status and found that the observed clusters were not related to the patient’s HPV status (Figure 2B) (average Silhouette score ~0.03). We performed a Wilcoxon rank–sum test to identify features that are robust between Toshiba and GE scanners (FDR ≥ 5%). We found that 53% (989 of 1874) of the radiomic features were significantly associated with scanner classification (FDR < 5%). We then computed the t-SNE clusters again using only the robust (FDR ≥ 5%) features and confirmed that the data did not cluster by scanner group (Figure S2). To illustrate the distribution of robust features, the average (over 100 separate runs) proportion of robust features according to the total number of features in each class and a total number of robust features were also estimated. On average 740 (±90) features (out of 1847) were significantly associated with the scanner manufacturer (FDR < 5%). The greatest number of robust features belonged to the GLCM group (24 ± 1.1%) when numbers were normalized to the total number of robust features (Figure 3A). However, when the number of robust features was normalized to the number of features in that class most of the GLDM and NGTDM (55%) features were robust against the scanner manufacturer (Figure 3C). Also for each group, the distribution of robust features after applying different image filters was compared to the original images (Figure S3). All feature groups showed improvements in the number of robust features after applying LoG, LBP and Wavelet features, implying that these filters could be of great importance in increasing feature robustness. The filter group with the largest proportion of robust features (the number of robust features normalized by the total number of features in that group) was the Exponential (86%), compared to original non-filter features (78%) (Figure 3B,D).

**Figure 4:**
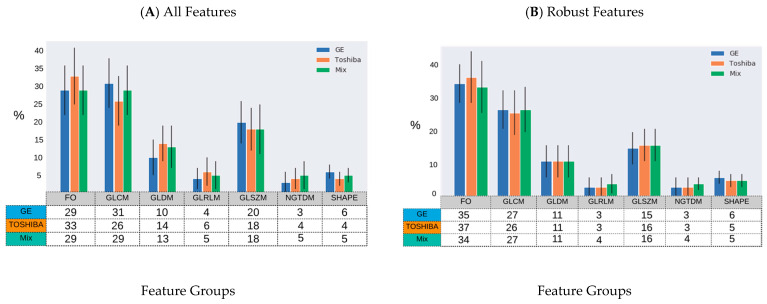

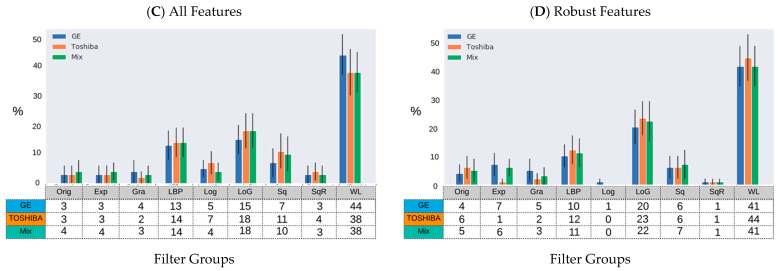
Percentage of HPV-relevant features for different samples (GE, Toshiba and mix) according to the type of feature group and imaging filters prior to robustness evaluation (**A**,**B**) and after (**C**,**D**). (**A**,**B**): GLRLM: Gray Level Run Length Matrix; GLSZM: Gray Level Size Zone Matrix; FO: First Order Statistics; GLCM: Gray Level Co-Occurrence Matrix; GLDM: Gray Level Dependence Matrix; NGTDM: Neighboring Gray Tone Difference Matrix. (**C**,**D**): Orig: Original; Exp: Exponential; Gra: Gradient; LBP: Local Binary Pattern; Log: Logarithm; LoG: Laplacian of Gaussian; Sq: Square, SqR; Square Root; and WL: Wavelet.

The distribution of the selected robust features deemed HPV-relevant (after mRMRE feature selection) is presented in Figure 4. This result showed that first order statistics (Figure 4A) and Wavelet filters (Figure 4C) give rise to the largest number of robust features among feature groups and filter groups respectively. However, after removing non-robust features, GLDM and NGTDM features comprise the largest group of HPV-relevant features (Figure 4B). However, Wavelet features were still the most HPV-relevant features even after removing non-robust features (Figure 4D).

**Figure 5:**
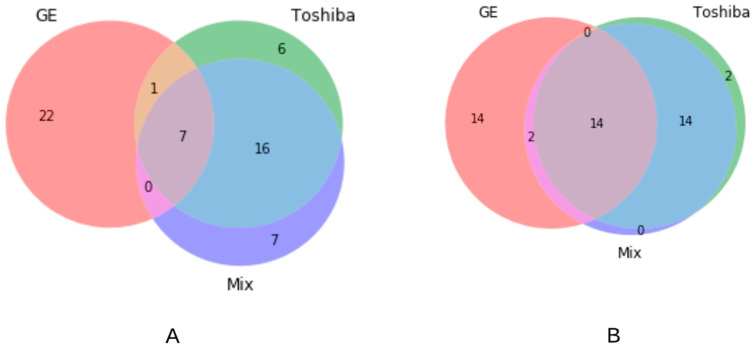
Venn diagram of the common radiomic features selected out of samples from different CT scanner types from (**A**) all radiomic features and (**B**) only robust features.

We also evaluated the number of common features selected from different groups (i.e., Toshiba, GE and Mix) out of all the available features (Figure 5A) and robust features (Figure 5B). As is shown in the Venn diagram (Figure 5), 7 (*p*-value < 10^−3^ features were found to be common across different scanners when all features were used for modelling. This number increased to 14 (*p*-value < 10^−3^) when only robust features were used. The number of common features between Toshiba–GE, Toshiba–Mix and GE–Mix was 1, 16, and 0 respectively when all features were used for feature selection and 0, 14, and 2 respectively when only robust features were applied. After removing non-robust features, the number of common features among all groups increased from 7 to 14 features.

### Scanner Grouping and Prediction of HPV Status

**Figure 6:**
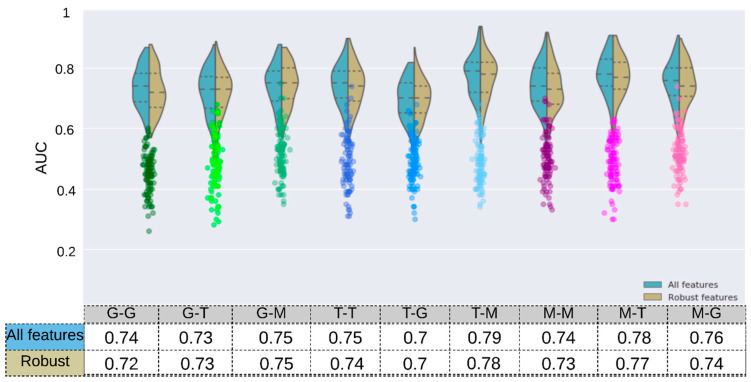
The prediction accuracy (AUC) of HPV status obtained by the RF Classifiers for 9 configurations of scanner manufacturers, used for training and tests after 100 runs. The Wilcoxon rank–sum test was applied to select robust features against the scanner models (adjusted *p*-value > 10^−2^, Bonferroni correction). The mRMRe was used to select HPV-relevant features. The model was trained and tested on different sets based on their scanner manufacturer (T: Toshiba, G: GE, M: mix) with a different number of features (mRMRe and mRMR + Robust). The corresponding scatter plots (color circles below each violin plot) are from the same model but with random dependent variables.

The highest and lowest median AUC values were 0.79 (*p*-value < 10^−4^) and 0.70 (*p*-value: 5.4 × 10^−3^) and obtained with the Toshiba–Mix and Toshiba–GE respectively (Figure 6 and Figure S4).

For models trained on one scanner manufacturer, the highest and lowest results in terms of median AUC were obtained when they were tested on the Mix sample (i.e., GE–Mix [0.75, *p*-value: 4 × 10^−4^], Toshiba–Mix [0.79, *p*-value < 10^−4^ ]) and other scanner manufacturers (i.e., GE–Toshiba [0.73, *p*-value: 7 × 10^−4^], Toshiba–GE [0.70, *p*-value: 5.4 × 10^−3^]) respectively.

The RF model was trained and tested on both samples (Mix) and reached a median training and validation AUC of 0.79 (*p*-value < 10^−4^) and 0.74 (*p*-value: 4 × 10^−4^) respectively. Furthermore, this model was trained on robust features (FDR ≥ 0.05) and reached a median AUC of 0.77 (*p*-value < 10^−4^) and 0.73 (*p*-value: 4 × 10^−4^) in training and validation respectively. This result reveals that robust features tend to reduce the difference between the training and validation AUC which can be best described as reduction in the models’ overfitting. Models trained on Mix but tested on one scanner manufacturer resulted in AUC values of 0.78 (*p*-value < 10^−4^) and 0.76 (*p*-value: 6 × 10^−4^) for Mix–Toshiba and Mix–GE models respectively.

The training AUC in all models decreased after removing non-robust features (GE: 0.80→0.77, Toshiba: 0.81→0.79, Mix: 0.79→0.77).

The models with single scanner manufacturer did not result in a significantly different AUC value (GE-GE: 0.74 (*p*-value < 10^−4^), Toshiba-Toshiba: 0.75 (*p*-value: 6 × 10^−4^)) compared to the models with both scanners (Mix–Mix: 0.74). After removing non-robust features, the Mix–Mix model reached a train and validation AUC of 0.77 (*p*-value < 10^−4^) and 0.73 (*p*-value: 4 × 10^−4^) respectively (Figure 6).

## 4. Discussion

Our goal was not to find a model that led to a good classification of HPV status but to assess the impact of different CT scanners on the prediction performance of the radiomic model. To do this, we assessed the effects of different scanner manufacturers on the robustness of radiomic features and their use for the prediction of HPV status in OPC patients, an increasingly common type of head and neck cancer. Although there are many studies investigating the robustness of radiomic features, few have reported the impact of feature robustness on the predictive performance of radiomic models. In this study, the scanner manufacturer affects the models’ accuracy in predicting HPV status using hand-engineered radiomics features.

Scanner dependency is an important aspect of radiomics research that has previously been evaluated in phantom studies [14,30]. In these studies, the researchers used CCR phantom images from different scanners by different manufacturers and concluded that most features have significant scanner dependency and pointed out the importance of minimizing this effect in future radiomics studies. Other studies highlighted that different CT scanners have been proven to have variation in their Hounsfield units even with the same acquisition parameters [31,32]. Perrin et al. showed that when they included all patients from all scanners, the number of liver tumor-derived robust features (concordance correlation coefficient > 0.9) from the same scanner model decreased from 75 to 35 (out of 254) [33]. This retrospective study evaluated the impact of scanner manufacturer on the prediction of HPV status using CT-derived radiomic features. To the best of our knowledge this is the first study evaluating scanner dependency using patient data.

To evaluate the effect of domain dependency on the prediction of HPV status, RF classifiers were trained and tested on samples from different scanners (GE vs Toshiba vs. Mix). A total of 1874 radiomic features were extracted from the GTV of 1294 OPC patients. The t-SNE clustering and the Wilcoxon rank-sum tests were then utilized to visualize the dependence of radiomic features on scanner manufacturers. This allowed us to quantitatively measure the statistical variation between features from each scanner manufacturer. The t-SNE clustering showed that radiomic features are dependent on the scanner manufacturer.

We found that most of the robust features belonged to the GLCM group, which was in accordance with previous studies [34,35]. In a study evaluating the variations of radiomic features extracted from 20 NSCLC patients from different scanners, Busyness and texture strength of the NGTDM class were the most and least robust features, respectively [14]. Based on the definition in [36], NGTDM textural features reflect the intensity differences between a voxel and its neighboring voxels. With the exception of Wavelet imaging, filters do not significantly change the distribution of robust features from the non-filtered images (Original). One reason behind the superiority of Wavelet filters could be the greater number of features (744 vs. 93) in this group compared to other groups which may overestimate the positive effects of this filter. However, Wavelet features have shown interesting applications in radiomics studies mostly because of their potential to highlight hidden texture information [37].

Finally, different combinations of samples from different scanner manufacturers (GE, Toshiba, and Mix) have been resampled to evaluate the effect of scanner manufacturer on the prediction of HPV status. We identified that the prediction model that yielded the best AUC (equal to 0.79) was the Toshiba–Mix configuration along with the use of all the radiomic features for training. Among all configurations, inverse models, the models trained and tested on two different scanner types (i.e., GE–Toshiba and Toshiba–GE), resulted in worst AUC values (0.73 and 0.7 respectively) which highlights the effects of scanner type on the prediction result. We also observed a lower inconsistency across models trained and tested on the same data set (i.e., GE–GE: 0.74, Toshiba–Toshiba: 0.75, Mix–Mix: 0.74). However, when restricted to a more clinically suitable condition, models trained on the Mix dataset and tested on one scanner (Mix–Toshiba, Mix–GE) saw an improved AUC value compared to inverse models, but not one as good as the best performing model (Toshiba–Mix). However, this finding is highly dependent on the clinical outcome of interest (i.e., HPS status), and is subject to change if other outcomes are going to be predicted. We also found a bias in the results in favor of one scanner manufacturer (Toshiba).

One interesting result of this study is that removing non-robust features reduced the accuracy of the predictions in all configurations. A hypothesis behind this might be that non-robust features are not necessarily irrelevant for the prediction of HPV status, and by removing them, the predictive model lacks enough learning information. Our assumption was that focusing on robust and HPV-relevant features might be more predictive than non-robust but HPV-relevant features. However, this finding shows that removing non-robust features does affect model performance and highlights the applicability of feature harmonization techniques like ComBat [38], providing it becomes applicable to upcoming samples.

The current study has multiple limitations. First, we did not have the same patients imaged in the two groups of scanners, which is the standard approach in this type of study; as a result we were not able to use the common reproducibility metric used in other similar studies for variables such as Intra-class correlation (ICC) [12], Concordance Correlation coefficient (CCC) [13], or Coefficient of Variation (COV) [39]. However, this is acceptable since we were dealing with real patient data, and it is not currently feasible to collect this number of samples (1294 patients) with HPV status and two sets of images from different scanner manufacturers. Another limitation was that the samples from one scanner (Toshiba) had undergone contrast agent administration while the other group were non-contrast examinations. Although the GTV area is a very small region, we believe that the contrast media administration is a major contributor to the clustering since it significantly affects the CT Hounsfield values and can variably change internal CT numbers within tumors by highlighting regions with more/less contrast uptake and/or vasculature. The effects of contrast enhancement have been studied in the delayed phase of CT images for NSCLC patients, which shows that radiomic features are substantially affected. Furthermore, the variability of radiomic features due to contrast uptake was found to be dependent largely on patient characteristics [40]. However, in this study, we focused on the effects of domain dependency on prediction performance, disregarding the exact differences between the domains.

## 5. Conclusions

In this study, the scanner manufacturer grouping affects prediction accuracy of HPV status using hand-engineered radiomics features. The optimal prediction accuracy was achieved when the training set included only one specific type of scanner (i.e., Toshiba) which reflects a bias in radiomic features owing to the scanner type and/or scanning methods used on that device. Furthermore, incorporating robust features neither improved predictions nor the robustness of radiomic models across different configurations. This result demonstrated the importance of imaging parameters, such as hardware parameters and protocols, for training radiomic-based classifiers. Future directions for this line of study include evaluating how this finding will translate into clinical applications of radiomic models and potential solutions such as feature harmonization to remove this scanner dependency.

## Data Availability

To ensure the full reproducibility of our study we created a Code Ocean capsule to allow users to easily run and reuse our analysis pipeline. The code for all of the computations and associated Code Ocean capsule are available upon reasonable request to corresponding author.

## Supplementary Materials

The following are available online at https://www.mdpi.com/article/10.3390/cancers13092269/s1, Figure S1: Distribution of patient base on demographic information (A) and the type of scanner manufacturer over time (B), Figure S2: t-SNE clusters: A: robust (Wilcoxon rank-sum test, *p*-value > 0.05, corrected for the number of features using Bonferroni method); B: non-robust features, Figure S3: The proportion of robust features with different image filters. Values were normalized to the total number of features in each category, Figure S4: Average Receiver Operating Characteristic (ROC) curve of different models over 100 separate runs. Models are built over the HPV relevant features regardless of the robustness (A) and robust features (Wilcoxon Rank-sum test) (B). The first part of the model name stands for the type of training set and the second part represents the type of test set. The average of the AUC values was shown inside parentheses.
