# Supplementary material for: Prediction of Human Papillomavirus (HPV) Association of Oropharyngeal Cancer (OPC) Using Radiomics: The Impact of the Variation of CT Scanner
Source: Cancers (Basel). 2021 May 8;13(9):2269. doi: 10.3390/cancers13092269 (PMC8125906; doi:10.3390/cancers13092269)
Supplement: Supplementary file 1 [file cancers-13-02269-s001.zip › cancers-1145948-supplementary.pdf]

cancers

Article

Prediction of Human Papillomavirus (HPV) Association of Oropharyngeal Cancer (OPC) using Radiomics: The Impact of the Variation of CT Scanner

Reza Reiazi, Colin Arrowsmith, Mattea Welch, Farnoosh Abbas-Aghababazadeh, Christopher Eeles, Tony Tadic, Andrew J. Hope, Scott V. Bratman and Benjamin Haibe-Kains

Supplementary Figures

A

**Age:**	*Male*	61 (22–91)
	Female	62 (21–89)
**HPV status:**	*Positive*	824 (~64%)
	Negative	470 (~36%)
**Scanner**	*Toshiba*	1026 (~79%)
	GE	268 (~21%)

B

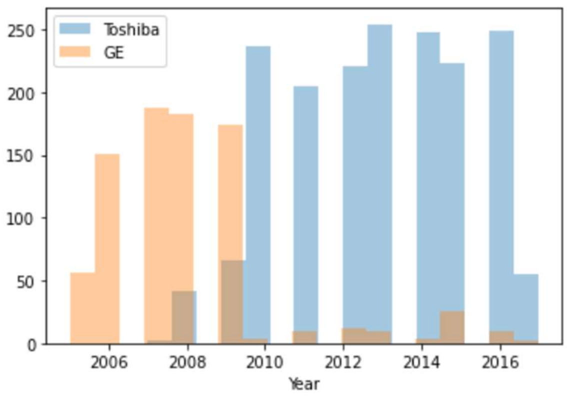

Figure S1. Distribution of patient base on demographic information (**A**) and the type of scanner manufacturer over time (**B**).

A B

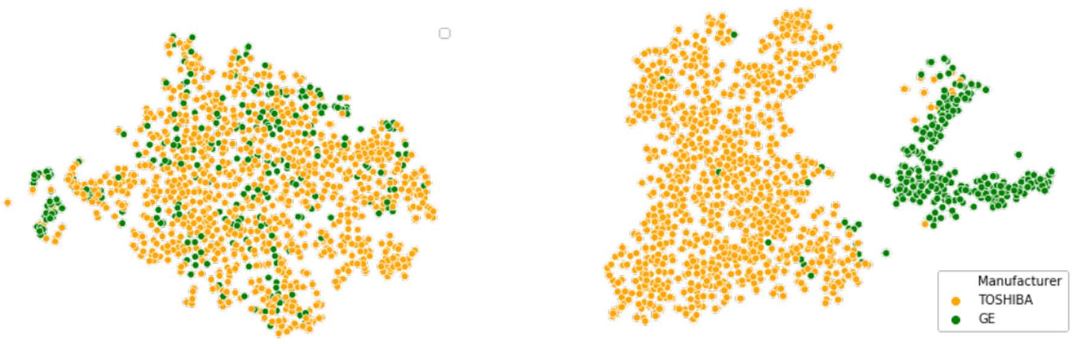

Figure S2. t-SNE clusters: (**A**) robust (Wilcoxon rank-sum test, p-value > 0.05, corrected for the number of features using Bonferroni method); (**B**) non-robust features.

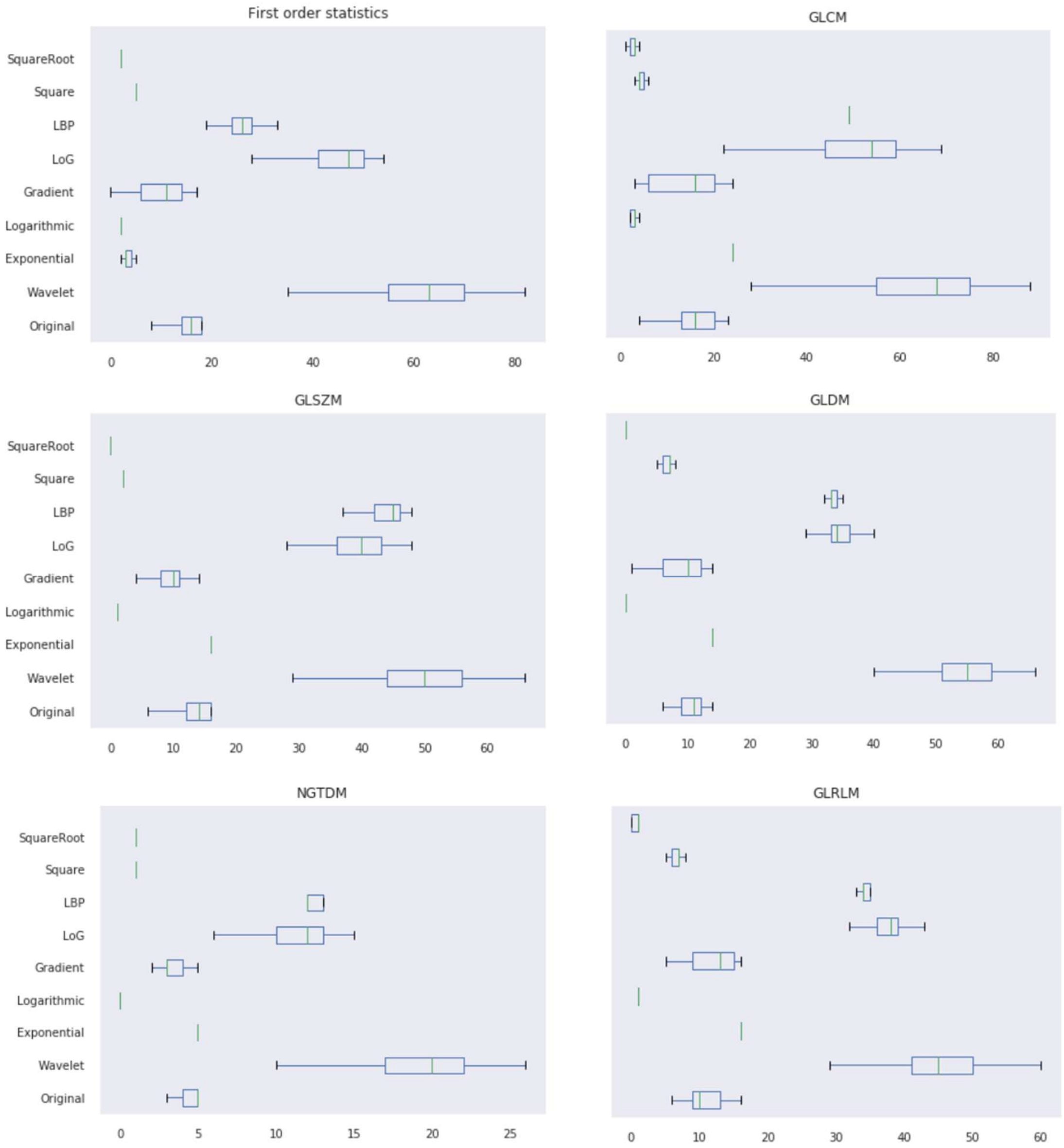

Figure S3. The proportion of robust features with different image filters. Values were normalized to the total number of features in each category.

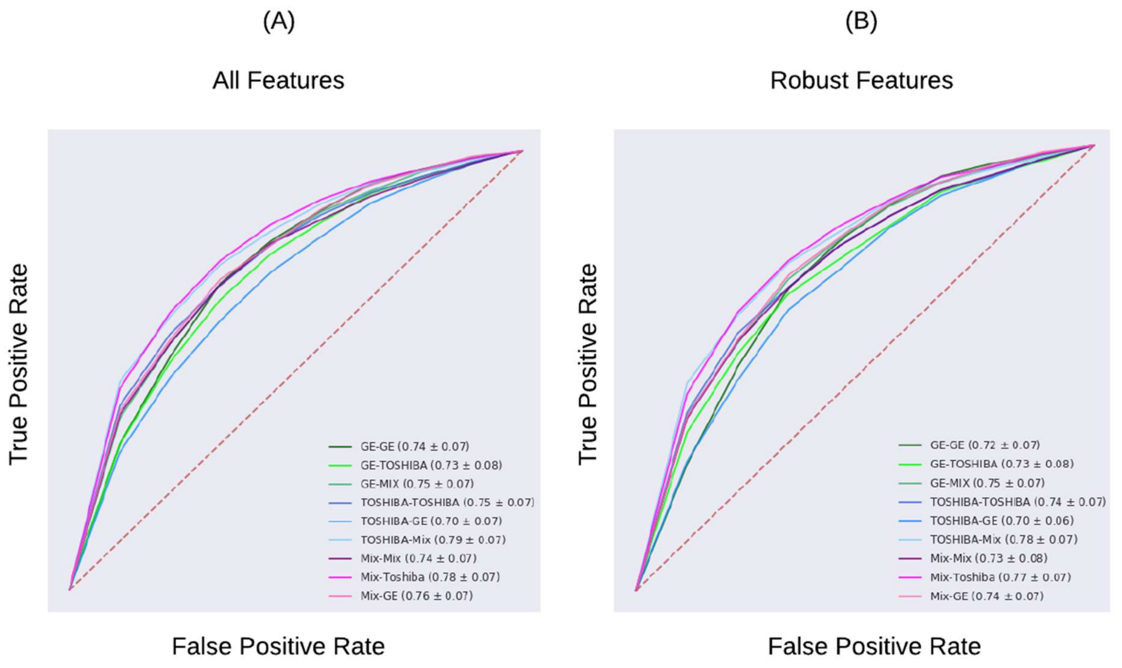

Figure S4. Average Receiver Operating Characteristic (ROC) curve of different models over 100 separate runs. Models are built over the HPV relevant features regardless of the robustness (**A**) and robust features (Wilcoxon Rank-sum test) (**B**). The first part of the model name stands for the type of training set and the second part represents the type of test set. The average of the AUC values was shown inside parentheses.
